# Study of Innovative GO/PBI Composites as Possible Proton Conducting Membranes for Electrochemical Devices

**DOI:** 10.3390/membranes13040428

**Published:** 2023-04-13

**Authors:** Matteo Di Virgilio, Andrea Basso Peressut, Angelo Pontoglio, Saverio Latorrata, Giovanni Dotelli

**Affiliations:** Department of Chemistry, Materials and Chemical Engineering “Giulio Natta”, Politecnico di Milano, Piazza Leonardo da Vinci 32, 20133 Milano, Italy

**Keywords:** polybenzimidazole, graphene oxide, composite membrane, ion exchange capacity, proton conductivity, self-assembling, proton exchange membrane, electrochemical device

## Abstract

The appeal of combining polybenzimidazole (PBI) and graphene oxide (GO) for the manufacturing of membranes is increasingly growing, due to their versatility. Nevertheless, GO has always been used only as a filler in the PBI matrix. In such context, this work proposes the design of a simple, safe, and reproducible procedure to prepare self-assembling GO/PBI composite membranes characterized by GO-to-PBI (X:Y) mass ratios of 1:3, 1:2, 1:1, 2:1, and 3:1. SEM and XRD suggested a homogenous reciprocal dispersion of GO and PBI, which established an alternated stacked structure by mutual π-π interactions among the benzimidazole rings of PBI and the aromatic domains of GO. TGA indicated a remarkable thermal stability of the composites. From mechanical tests, improved tensile strengths but worsened maximum strains were observed with respect to pure PBI. The preliminary evaluation of the suitability of the GO/PBI X:Y composites as proton exchange membranes was executed via IEC determination and EIS. GO/PBI 2:1 (IEC: 0.42 meq g^−1^; proton conductivity at 100 °C: 0.0464 S cm^−1^) and GO/PBI 3:1 (IEC: 0.80 meq g^−1^; proton conductivity at 100 °C: 0.0451 S cm^−1^) provided equivalent or superior performances with respect to similar PBI-based state-of-the-art materials.

## 1. Introduction

Polybenzimidazole (PBI) is a linear, non-perfluorinated, fully aromatic, heterocyclic polymer characterized by exceptional chemical resistance to acids and bases, high thermo-oxidative stability, high glass transition temperature (425–436 °C), good mechanical properties, and insulating features [1,2,3,4,5]. It is typically synthesized through condensation polymerization of 3,3′-diaminobenzidine (DAB) and isophthalic acid (IPA) with a 1:1 molar ratio in the presence of polyphosphoric acid (PPA), but other routes such as microwave-assisted organic synthesis and casting from methane sulphonic acid are feasible as well. This simplicity of production allows for ad hoc modifications of the polymer backbone by employing monomers with a distinct chemical nature. As a consequence, the controlled modulation of the physicochemical features of PBI, such as solubility, basicity, and film processing, is accessible. For instance, the distribution of nitrogen atoms within the polymeric chains fulfills a key role in the definition of the global properties of the product [1,2]. A further possibility to enhance some specific characteristics of PBI is crosslinking, whose main goal is to improve the mechanical strength. Many different methodologies have been reported in the literature for an effective crosslinking. The most important ones are chemical and thermal treatments, promoting the generation of covalently, ionically, or mixed crosslinked structures [2,3,6,7,8].

PBI is mainly applied in membrane form in a wide range of very different fields. Depending on the specific tasks the membrane must accomplish, PBI can be combined with other materials to carefully tailor the overall features. Doping by means of phosphoric acid (PA) can be seen as the primary and most common epitome of combination [9,10,11], also due to its simplicity. The electron-acceptor –NH– moieties of the benzimidazoles, i.e., bicyclic groups consisting of fused benzene and imidazole rings, are able to readily tether PA to form single-phase doped proton exchange membranes (PEMs) with improved proton conductivity [4,6]. Examples of applications are nanofiltration [12,13], gas separation [14,15], flow batteries [16,17], fuel cells [11,18,19,20], and electrolyzers [21,22,23]. Flow batteries are promising devices for stationary energy storage characterized by environmental friendliness, design flexibility, extended lifecycle, and fast response times [24]. PEM fuel cells are currently considered as attractive power sources due to their high efficiency, compactness, modularity, and operations with theoretically zero emissions [25]. PEM water electrolyzers are among the preferred technologies for the production of highly pure hydrogen from renewable energy, and they display similar features to PEM fuel cells [26]. In all such cases, the PEM must fulfill a crucial task, and its performance strictly influences the overall outputs of the device in which it is employed.

The fabrication of PBI-based composites is appreciated as well, as demonstrated by the broad literature covering this topic and the numerous different materials discussed within it [27,28,29,30,31,32]. Among the spectrum of possible composite membranes, the ones combining PBI and graphene oxide (GO) [5,18,23] or its acid-functionalized derivatives, such as sulphonated GO (SGO) [33,34,35] and phosphonated GO (PGO) [35,36,37], appear to be promising for proton conduction applications. The reason why these materials are capturing so much attention to be used as organic fillers in this kind of membrane is explained by their wide set of strong points: electronic insulation, amphiphilic behavior, outstanding self-assembling ability, and two-dimensional structures decorated by a large number of oxygenated functional groups (carbonyl, hydroxyl, carboxyl, and epoxy groups, plus sulphonic acid and phosphonic acid groups in SGO and PGO, respectively) able to potentially guarantee good proton conductivity. Üregen et al. [18] prepared PA-doped PBI/GO composite membranes with loadings of GO equal to 2 and 5 wt%. The characterization results highlighted a good distribution of GO in the polymer, lower acid uptake but higher leaching levels at the highest GO content, optimal chemical stability for 2 wt% of GO, and improved proton transport properties. Sulaiman et al. [35] introduced GO, SGO, and PGO as fillers in PA-doped PBI membranes, studying mass contents of 0.25, 0.5, and 1 wt%. Their work helped to observe the good thermal stability of the composites, slightly reduced acid doping in the presence of the GO-based fillers, and enhanced proton conductivity with respect to bare PA-doped PBI, especially in the case of SGO and PGO.

Nevertheless, it is important to note two aspects about the GO/PBI composite membranes reported in the literature. The first is the use of GO only as a filler, never exceeding a content of 5 wt%. The second is the necessity of phosphoric acid doping regardless of the presence of GO in order to reach acceptable levels of proton conductivity. However, issues of migration of PA inside the membrane electrode assembly (MEA) and subsequent sharp reduction of proton conductivity, undesired acid leaching, and worsened mechanical properties have still to be reckoned with [10,22,27,28]. Therefore, a suitable answer to fill this research gap could be truly advantageous.

Within this frame, the present work sets out to a three-fold aim: (i) to develop a simple, safe, and reproducible preparation protocol for stable, self-assembling GO/PBI composite membranes characterized by high contents of both constituents; (ii) to study if the positive features of each component could be blended in a single material, hence to assert the viability of combining large relative quantities of GO and PBI; (iii) to preliminarily verify the eligibility of the as-prepared composite materials, i.e., without PA doping, for potential applications in proton conduction-based electrochemical devices working at temperatures belonging to the so-called “conductivity gap” [4], i.e., between 80 and 120 °C. To fulfill such purposes, five different membranes with GO-to-PBI mass ratios of 1:3, 1:2, 1:1, 2:1, and 3:1, corresponding to GO mass percentages one order of magnitude higher than those disclosed in the literature, were successfully fabricated. The set of samples was extensively investigated by means of scanning electron microscopy (SEM), X-ray diffraction (XRD), thermogravimetric analysis (TGA), tensile tests, evaluation of the ion exchange capacity (IEC), and electrochemical impedance spectroscopy (EIS). The direct comparison with benchmark pure GO and pure PBI membranes was exploited to certify the effectiveness of the designed preparation method and to infer how and the extent to which the two constituents affected the final properties of the GO/PBI composites.

## 2. Materials and Methods

### 2.1. Materials

Raw polybenzimidazole powder (trade name Dapozol^®^ PBI, chemical structure (C_20_H_12_N_4_)_n_) was provided by Blue World Technologies ApS (Aalborg, Denmark). It had an average molecular weight of approximately 93,000 g mol^−1^ and a density equal to 1.30 g cm^–3^ at 23 °C. It was characterized by a wool-like texture and a yellowish-brown color.

Graphene oxide was purchased as a viscous aqueous dispersion (concentration of 4 mg mL^−1^) from Graphenea, Inc. (Cambridge, MA, USA). The main properties were an average particle size of <10 µm, a monolayer content larger than 95%, and a pH of about 2.5.

Pure dimethyl sulfoxide (DMSO, chemical structure C_2_H_6_OS) was acquired from Sigma-Aldrich Corporation (St. Louis, MO, USA) in liquid form to be used as the solvent of the designed preparation procedure, described in Section 2.2. The same company also supplied pellets of sodium chloride (NaCl, ACS reagent, purity of ≥99.0%) and sodium hydroxide (NaOH, ACS reagent, purity of ≥97.0%).

### 2.2. Composite Membranes Preparation

**Figure 1:**
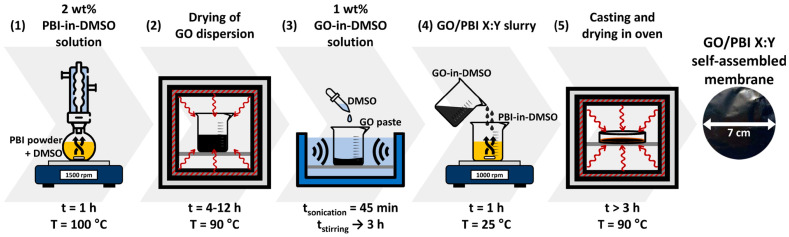
Preparation framework of the GO/PBI X:Y composite membranes.

The procedure to prepare the GO/PBI composite membranes was designed on the basis of solution casting techniques described in the literature [5,18,23]. It was composed of five different steps, reported in Figure 1, which are explained hereafter in detail.

The first task (1) to be accomplished was the thorough dissolution of the commercial PBI powder in an adequate organic solvent, and DMSO was chosen for such a purpose. A setup based on a round-bottomed flask immersed in a heated oil bath, connected to a reflux condenser, and placed upon a magnetic stirrer was implemented to prepare a 2 wt% PBI-in-DMSO solution. The operating conditions that guaranteed the complete dissolution of the PBI powder in the solvent were a temperature of 100 °C, a mixing rate of 1500 rpm, and a duration of 1 h. The second step (2) regarded the separation of GO from its dispersion medium, which was achieved by drying in oven at 90 °C. This phase lasted within 4 to 12 h, in order to ensure the almost complete evaporation of water and the obtainment of a pure GO paste. In the third step (3), DMSO was added to GO with the aim of producing a 1 wt% GO-in-DMSO solution. The system was homogenized via 45 min of ultrasonication, in the presence of ice to mitigate the temperature increase, followed by up to 3 h of magnetic stirring at 1500 rpm. In the fourth step (4), the prepared PBI-in-DMSO and GO-in-DMSO solutions were combined by magnetic mixing at 1000 rpm and room temperature for 1 h to attain a homogeneous GO/PBI slurry. In the last step (5), the slurry was slowly casted onto a 7 cm Petri dish; then, it underwent oven-drying at 90 °C for a minimum of 3 h. In this fashion, total removal of the solvent and self-assembly of the composite membrane were promoted. The GO/PBI membrane was in the end retrieved as an intact product by simple detachment from the Petri dish.

GO-to-PBI mass ratios of 1:3, 1:2, 1:1, 2:1, and 3:1 were studied in this work to verify the feasibility of preparing stable self-assembling membranes with high mass contents of either component, one order of magnitude higher than those typically found in the literature for GO [5,18,23,35]. Accordingly, the obtained samples were denominated GO/PBI X:Y, in which X and Y represent the GO and PBI terms, respectively, within the specific mass ratio characterizing the sample.

For the sake of completeness, pure GO and pure PBI membranes were also prepared to be considered as references. The pure GO membrane was obtained by ultrasonication of 37.50 mL of commercial dispersion for 1 h, vacuum-filtration on a polyvinylidene fluoride disk placed inside a 7 cm Büchner funnel for 24 h, and oven-drying of the deposit at 40 °C to finish the self-assembly. The pure PBI membrane was directly prepared from the 2 wt% PBI-in-DMSO solution, which was casted onto a 7 cm Petri dish and oven-dried for 3 h to foster the solvent evaporation and the formation of the pristine polymeric membrane.

Table 1 shows the quantities of GO, PBI, and corresponding solutions employed to fabricate the five different composite membranes and the two benchmark samples, as well as their average thickness values.

### 2.3. Characterization Techniques

#### 2.3.1. Scanning Electron Microscopy and X-ray Diffraction

The GO/PBI X:Y composite membranes were inspected via the scanning electron microscope (SEM) model EVO 50 EP (Carl Zeiss S.p.A., Oberkochen, Germany). Operating conditions were a chamber pressure of 10^–5^ Pa, an accelerating voltage of 20 kV, and a current probe of 20 mA.

X-ray diffraction (XRD) analysis was performed by means of the diffractometer model D8 Advance by Bruker Corporation (Billerica, MA, USA), which employed a Cu-K_α_ filament as the source of X-rays with wavelength (λ) equal to 0.154 nm, and a monochromator to filter them. A scanning rate of 0.02° per second with a count time of 1 s was applied in the 5–30° angular interval (2θ). Bragg’s Law (Equation (1)) allowed for computation of the corresponding interplanar distances d (nm):

(1) $$d\;\left(\mathrm{nm}\right)=\frac{\lambda }{2\sin\theta }$$

#### 2.3.2. Thermo-Mechanical Characterization

Investigation of the fabricated GO/PBI X:Y composite membranes from the thermal and mechanical standpoints was executed via thermogravimetric analysis (TGA) and tensile tests, respectively.

Concerning the former, TGA experiments were carried out through the EXSTAR 6000 TG/DTA 6300 by Seiko Instruments Inc. (Chiba, Japan). The samples underwent a temperature increase from 30 to 900 °C, which was controlled by applying a heating ramp of 10 °C min^−1^. An inert atmosphere was guaranteed by continuously providing a pure nitrogen stream of 55 mL min^−1^.

Regarding the latter, the Synergie 200 test system by MTS Systems Corporation (Eden Prairie, MN, USA) was used to determine the mechanical properties of the GO/PBI X:Y samples. A tensile mode with a strain rate of 1 mm min^−1^ was applied on four rectangular strips (70 mm in length and 10 mm in width) previously trimmed from each prepared membrane. Accordingly, information about tensile strength, maximum strain, and Young’s modulus of the composites was extrapolated from the corresponding stress–strain plots.

#### 2.3.3. Functional Characterization

Ion exchange capacity (IEC) is a parameter estimating the total active sites responsible for ion exchange per unit mass of a material, and it is typically measured in milliequivalents per gram (meq g^−1^). Acid–base back-titration is the main technique through which IEC can be assessed [38]. In this work, the methodology used for the determination of the IEC of the GO/PBI X:Y composite membranes involved at first a preliminary 1-h drying in oven at 60 °C of the samples, so as to assess their dry masses. Then, the samples were immersed in aqueous solutions of NaCl (concentration of 2 mol L^−1^, volume of 250 mL) for 48 h at room temperature to let the exchange between protons and Na^+^ ions occur. At last, the post-exchange NaCl solutions were titrated against controlled volumes of a NaOH solution (concentration of 0.01 mol L^−1^), and the corresponding titration curves were outlined. Defining V_NaOH_ (mL) as the turning point volumes graphically derived from such curves, C_NaOH_ (mmol mL^−1^) as the concentration of the titrating solution, and m_dry_ (g) as the measured masses of the dried samples, the IEC values were calculated according to Equation (2):

(2) $${\mathrm{IEC}\;(\mathrm{meq}\;g}^{-1})=\frac{V_{\mathrm{NaOH}}\cdot C_{\mathrm{NaOH}}}{m_{\mathrm{dry}}}$$

Additionally, the proton conductivity of the as-prepared GO/PBI X:Y composite membranes was investigated to preliminarily check their suitability for potential applications in proton exchange membrane electrochemical devices. To do so, electrochemical impedance spectroscopy (EIS) experiments were executed at four different temperatures (60, 80, 100, 120 °C) under humid conditions. The equipment involved for such experiments included:

- a lab-built chamber filled with 0.6 L of deionized water and equipped with an external shell in which heated oil circulated;
- a thermocouple for the temperature control;
- two stainless steel electrodes assembled on a Teflon^®^ cell inside which the samples were clamped [39];
- a STEMlab™ 125–14 Bode Analyzer by Red Pitaya (Solkan, Slovenia).

Three EIS tests were performed for each membrane on previously cut rectangular samples (35 mm length × 10 mm width), in order to guarantee reliable results. The samples were initially dried at 60 °C for 2 h to measure their dry thicknesses. Then, they were fastened to the Teflon^®^ cell and inserted in the humid chamber, where they were left for 1 h. After this time, the Bode Analyzer was set up to a potentiostatic mode (signal amplitude of 1 V), and the samples were subjected to EIS tests in the 1–10^7^ Hz frequency interval. The outcomes were Bode diagrams, which were converted into Nyquist plots to be fitted by means of the ZView^®^ software 3.0 (Scribner Associates Inc., Southern Pines, NC, USA). The aim of the fitting was the evaluation of the internal resistance R_i_ (Ω), one of the elements of the equivalent circuit chosen to describe the studied electrochemical system [40]. Values of proton conductivity σ (S cm^−1^) were extrapolated as the inverse of the resistivity ρ (Ω cm) according to Equation (3), in which w (cm) and t (cm) are the widths and thicknesses of the samples, respectively, and d (cm) is the spacing between the electrodes:

(3) $${\sigma \;(S\;\mathrm{cm}}^{-1})=\frac{1}{\rho \;(\Omega \;\mathrm{cm})}=\frac{d}{w\cdot t\cdot R_{i}}$$

## 3. Results and Discussion

### 3.1. Composite Membranes

The fundamental goal of this work was the design of a well-defined set of steps for the preparation of GO/PBI composite membranes with high mass contents of both components, i.e., from a 1:3 mass ratio, corresponding to a minimum GO content of 25 wt%, to a 3:1 mass ratio, corresponding to a maximum GO content of 75 wt%. The procedure was desired to be as simple, safe, inexpensive, and reproducible as possible. Solution casting was considered to be the method best fulfilling such requirements, although the authors had to address some critical points. For instance, several works relied on the use of N,N-dimethylacetamide (DMAc) [18,20,30,36] or N,N-dimethylformamide (DMF) [37,41,42] as organic solvents for PBI. Nevertheless, these substances are renowned for their intrinsic dangerousness toward human safety, inasmuch they are toxic and irritating. Therefore, the authors opted for DMSO since this non-toxic, highly polar substance is considered as an overall less hazardous diluent [43].

Another important issue encountered in this work was the incompatibility between the commercial GO aqueous dispersion and the prepared PBI-in-DMSO solution. Their direct combination by magnetic mixing caused a fast separation of small aggregates of PBI, which prevented the formation of a homogenous slurry and, consequently, the fabrication of self-assembling composite membranes. Therefore, drying of the GO aqueous dispersion and dissolution of GO in DMSO were mandatory to guarantee chemical compatibility between the two solutions. In this fashion, the authors aimed at guaranteeing the reproducibility of the proposed procedure.

Particular attention was also given to the selection of the process temperatures. In step (3) of Figure 1, drying of the GO aqueous dispersion was performed at 90 °C to avoid an unwanted thermal reduction of GO and resulting loss of oxygen-bearing functionalities, which previous studies identified to occur slightly above 100 °C [44]. The same concept was applied in step (5) to preserve GO in the composite membranes while allowing for a slow and controlled evaporation of DMSO [45]. As a direct effect, different durations were required for the production of each GO/PBI X:Y composite membrane. Specifically, step (3) lasted a minimum of 4 h up to a maximum of 12 h depending on the initial amount of GO aqueous dispersion needed to fabricate one sample rather than another, i.e., 12.5 mL (0.05 g of GO) and 37.5 mL (0.15 g of GO) of commercial product to be dried, respectively. Step (5) required at least 3 h in the specific case of GO/PBI 1:3 (12.50 g of slurry), whereas solvent evaporation was protracted for higher quantities of casted slurry.

**Figure 2:**
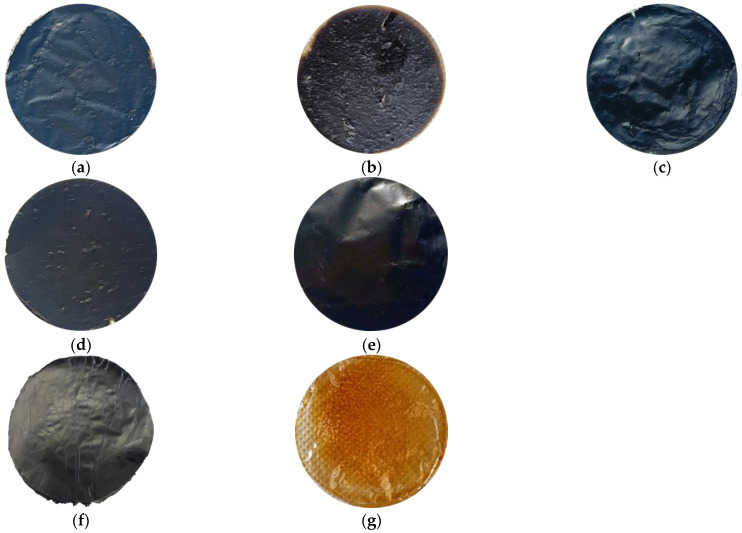
Macroscopic appearance of the prepared membranes: (**a**) GO/PBI 1:3; (**b**) GO/PBI 1:2; (**c**) GO/PBI 1:1; (**d**) GO/PBI 2:1; (**e**) GO/PBI 3:1; (**f**) pure GO; (**g**) pure PBI.

Due to the polymeric nature of PBI and to the high tendency of GO to self-assemble, preparation of the composite membranes by means of the designed protocol delineated in Section 2.2 was easily reproducible. Several stable samples were obtained for each of the studied mass ratios for characterization aims. The macroscopic appearances of the GO/PBI X:Y composites, pure GO, and pure PBI are displayed in Figure 2. The composites were characterized by an overall homogeneity, without any visible damage. A slightly rougher and more wrinkled surface was observed with respect to pure GO and pure PBI, especially at GO mass contents of 33, 50, and 67 wt%. Furthermore, the ability of the two components to coexist in a uniform single material was preliminarily suggested, in a qualitative way, by a sort of color gradient among the composite membranes. The samples containing more GO, black by nature (Figure 2f), tended to be very dark. On the contrary, the membranes with a larger presence of PBI, whose peculiar color is yellowish-orange (Figure 2g), were brighter.

### 3.2. Composite Membranes Morphology and Microstructure

**Figure 3:**
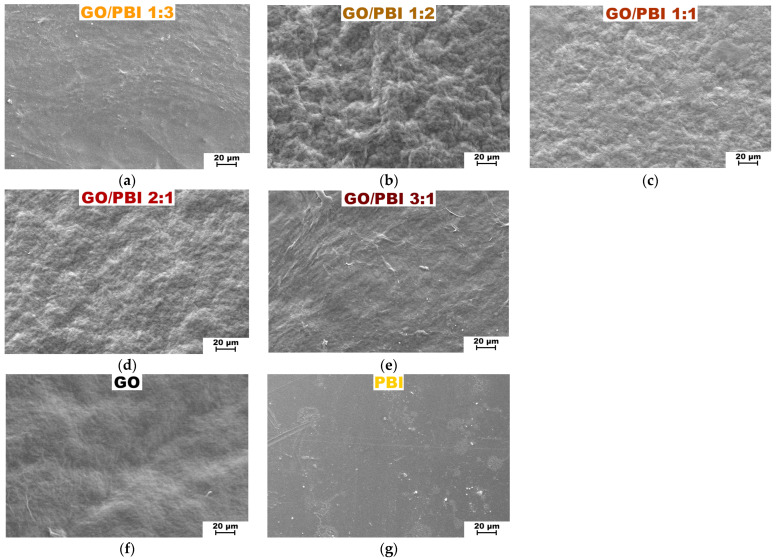
SEM images at 1000× magnification of the composite membranes: (**a**) GO/PBI 1:3; (**b**) GO/PBI 1:2; (**c**) GO/PBI 1:1; (**d**) GO/PBI 2:1; (**e**) GO/PBI 3:1. SEM images at 1000× magnification of (**f**) pure GO membrane and (**g**) pure PBI membrane are reported for comparison.

Scanning electron microscopy 1000× top views of the fabricated GO/PBI X:Y membranes are displayed in Figure 3, together with the ones of the reference GO and PBI samples. SEM analysis confirmed the morphological uniformity of all the investigated membranes. The absence of any significant defect or distortion could be imputed to the reliability of the fabrication procedure, which appeared to be consistent for whichever of the employed mass ratios. Nonetheless, the addition of increasing GO contents caused the surface of the corresponding composites to become more wrinkled. The more evident roughness was registered for GO/PBI 1:2 (Figure 3b), GO/PBI 1:1 (Figure 3c), and GO/PBI 2:1 (Figure 3d). This observation could be interpreted by the balance of internal forces acting within the bulk of the membranes. Hydrogen bonds among the GO flakes increased their tendency to cluster, but at the same time, π-π interactions could be established among the benzimidazole rings of the polymer strands and the multiple sp^2^-hybridized domains of GO [2,20,23,46]. These two contributions could disturb the generation of planar surfaces, therefore being responsible for the coarseness visible throughout these three samples [18,35,39]. On the contrary, GO/PBI 1:3 (Figure 3a) and GO/PBI 3:1 (Figure 3e) seemed to deviate from this pattern. The former exhibited an overall smooth surface without any pinholes, bubbles, or cracks. It demonstrated to be very similar to the pristine PBI membrane (Figure 3g) [18,29,30,33], as expected for the composite characterized by the highest mass content of PBI. In a perfectly specular way, the latter displayed a moderately wavy surface akin to the bare GO membrane (Figure 3f), coherently with the highest GO concentration. The thin ridges visible on the surface of GO/PBI 3:1 could presumably be small GO sheets that wrinkled out of the sample during the oven-drying step. Nevertheless, the establishment of less GO-PBI mutual interactions and more PBI-PBI (for GO/PBI 1:3) and GO-GO (for GO/PBI 3:1) interactions can explain the discussed outcomes.

In general, no evident macroscopic agglomerations were observed for the studied mass ratios, underlining the effectiveness of the dissolution of both PBI and GO in DMSO and their mixing to produce a homogeneous slurry. The observed uniformity and the absence of unwanted large agglomerations may be taken as a proof of cohesion among the GO flakes and the PBI polymeric chains at the microscopic level, exerted by mutual π-π interactions. These conditions can be considered as ideal for the production of stable composite membranes.

**Figure 4:**
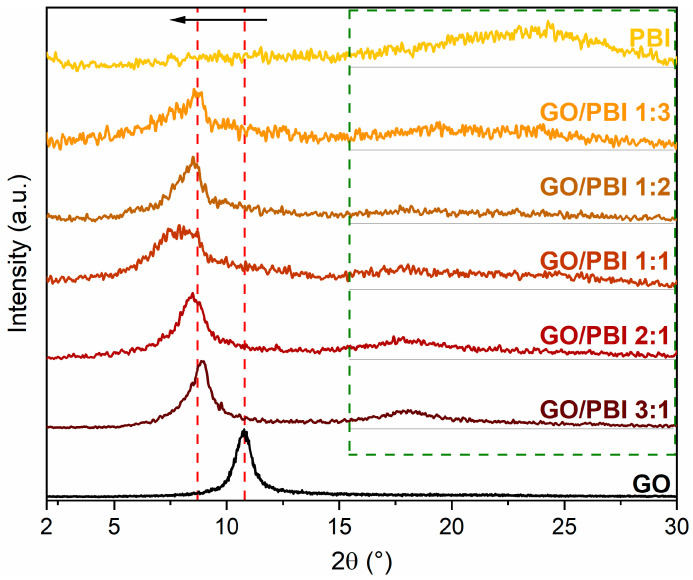
X-ray diffraction patterns of the GO/PBI X:Y composites, pure GO, and pure PBI membranes.

The diffractograms of the GO/PBI X:Y composite membranes, pure GO, and pure PBI are shown in Figure 4, whereas the interplanar distances calculated for the fundamental reflections are reported in Table 2. Before proceeding with the analysis of the composite samples, it is essential to describe the XRD patterns acquired for pristine GO and PBI. A narrow and well-defined reflection centered at 2θ equal to 10.78°, corresponding to an interplanar distance of 0.82 nm, was observed in the GO diffractogram. Such a feature agreed with the well-known reflection of crystalline GO, related to its 001-diffraction plane. It is typically attributed to the influence of the multiple hydrophilic oxygenated functionalities present on the GO carbonaceous backbone and to the associated intercalation of water molecules, both responsible for an interplanar distance between the GO layers that is more than doubled with respect to virgin graphene planes (002-diffraction plane, 2θ equal to 26.60°, interplanar distance of 0.34 nm) [18,33,39,47].

PBI membrane displayed a broad reflection ranging between 2θ values of 20 and 30°, approximately centered at 24.78°. The semi-crystalline regions of PBI were identified as the primary reason for this outcome. However, the broadness and rather low intensity of the reflection indicated the predominance of amorphous domains within the polymer. The calculated interplanar distance was 0.37 nm, equivalent to the spacing between two parallel, face-to-face packed benzimidazole chains and comparable with that of various heterocyclic polymers [28,32,37,48,49].

Two key points can be elucidated from the analysis of the diffractograms of the GO/PBI X:Y composites. First and foremost, the GO-related reflection shifted toward smaller diffraction angles, between 7.88 and 8.88°, in all the as-prepared membranes (red dashed lines). The interplanar distances extrapolated from such 2θ values ranged from 0.99 to 1.12 nm. The preservation of the oxygen-containing moieties of GO was confirmed by such a result, excluding thermal reduction during the fabrication process. Moreover, the increased patterns’ smoothness at higher GO contents could suggest the dominance of GO in establishing a slightly more ordered and, hence, more crystalline structure. The increase in the interplanar distance could probably be explained by the intercalation of PBI strands amidst adjacent GO layers, fostered by the development of preferential π-π interactions among the aromatic regions of both constituents. The affinity between GO and PBI may be further confirmed by the left-shift of the GO reflection even when the content of PBI was the lowest, i.e., in GO/PBI 3:1. A slight discrepancy with such interpretation was represented by the sample containing the same mass of both GO and PBI, i.e., GO/PBI 1:1, which exhibited an anomalously higher interplanar distance (1.12 nm) with respect to the other composite membranes (0.99–1.04 nm). A possible explanation might be found in the specific composition of this specimen, characterized by the total absence of disproportion between GO and PBI. Unlike in the other composites, such balance between the two components could give rise to different mutual interactions, which may cause an unexpected effect on the stacking of GO layers with PBI. A more thorough investigation of this aspect should be considered in forthcoming works.

The second observation concerned the less evident and very broad contribution visible at 2θ values within the 15–30° range (green dashed frame), completely absent in the diffractogram of pristine GO. This outcome again recommended the reciprocal influence of the two components, considering the shift toward smaller diffraction angles and therefore larger interplanar distances, of the broad reflection typical of semi-crystalline PBI. The insertion of GO flakes in the middle of adjacent polymeric chains could be a reasonable justification, strengthened by the rising of the PBI-related reflection even at the lowest GO content, i.e., in GO/PBI 1:3.

The XRD analysis supported the assumption, previously made by analyzing the SEM images, of an effective mixing of the two components during the designed preparation procedure. Furthermore, it allowed to suppose how the mutual interfacial interactions helped the alignment of GO flakes and PBI strands in an alternated stacked framework (Figure S1) for whichever investigated mass ratio.

### 3.3. Thermal and Mechanical Properties

**Figure 5:**
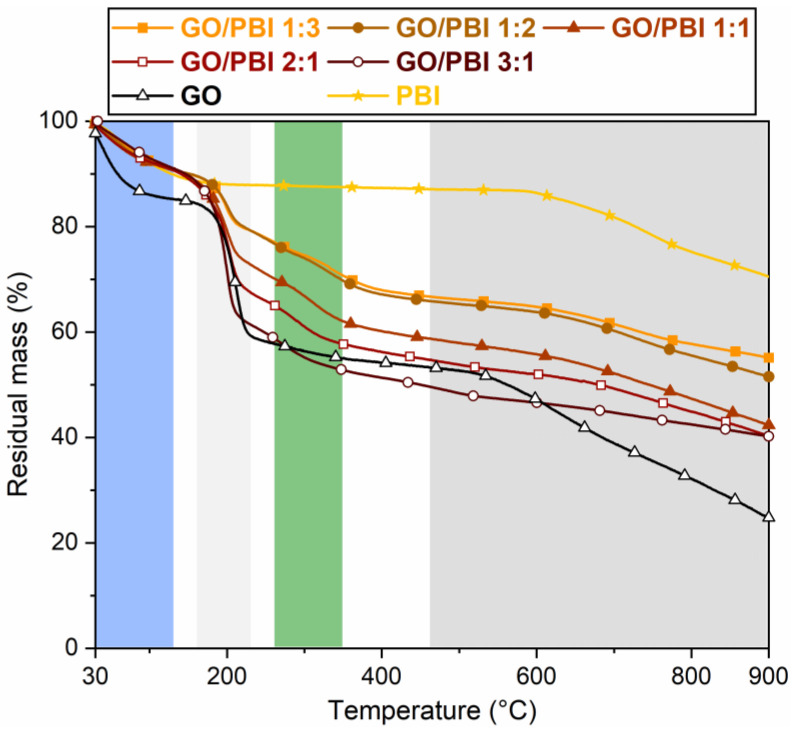
Thermograms of the GO/PBI X:Y composites, pure GO, and pure PBI membranes.

According to the thermograms highlighted in Figure 5, the GO/PBI X:Y composite membranes were characterized by an overall four-stage mass loss, pure GO by a three-stage mass loss, and pure PBI by a two-stage mass loss. The one occurring up to 130 °C (blue) was observed for all the studied materials, albeit with different extents. It was equal to ≈9–10% for the composites and PBI, and ≈15% for GO. This mass loss was related to the evaporation of the physically adsorbed water molecules within the membranes. In the case of the GO/PBI X:Y samples, moisture retained by the GO paste obtained in step (2) of the designed procedure was supposed to be the mainly accountable factor. The removal of traces of residual solvent still present after step (5) could have partially occurred in this range as well [34,38], although its contribution could be considered as negligible. The compatibility between GO and PBI appeared to have a significant influence on the thermal properties of the composite membranes. They were able to better maintain trapped moisture with respect to bare GO, guaranteeing a mass drop similar to virgin PBI. This result could be explained by assuming that the water retention of the hydrophilic GO groups was improved in the alternated stacked structure promoted by the combination with PBI chains.

The second stage (light grey), detected roughly between 160 and 220 °C for the composites and pure GO, was more prominent than the first one. It was ascribed to the decomposition of the oxygenated functional groups within GO [37,38]. Larger mass drops were measured while the GO mass content increased in the composites. To clarify this aspect, GO/PBI 1:3, which had the lowest GO content, exhibited a 9.7% mass loss. On the other hand, GO/PBI 3:1, which had the highest GO content, displayed a 26.2% mass loss, as expected comparable to that of bare GO (22.1%).

The third mass loss (green) between 270 and 350 °C, typical of the GO/PBI X:Y membranes only, was slow and distributed over the entire temperature range. It may be associated with the decomposition of the –NH– and –N= functional groups of the imidazole rings [30]. The absence of this drop in the pure PBI thermogram could be due to the interaction among PBI strands and GO layers, which might have made the polymer slightly more prone to thermal degradation.

In the end, the fourth stage (dark grey) started at approximately 500 °C. It was related to the breakdown of the GO framework [38], to which the contribution of the partial decomposition of the PBI carbonaceous backbone was added at 600 °C [16,29,49]. Pure PBI demonstrated the largest residual mass at 900 °C (70.5%), such that even the lowest polymer content in GO/PBI 3:1 permitted to have a 15%-heavier residue with respect to pure GO.

**Figure 6:**
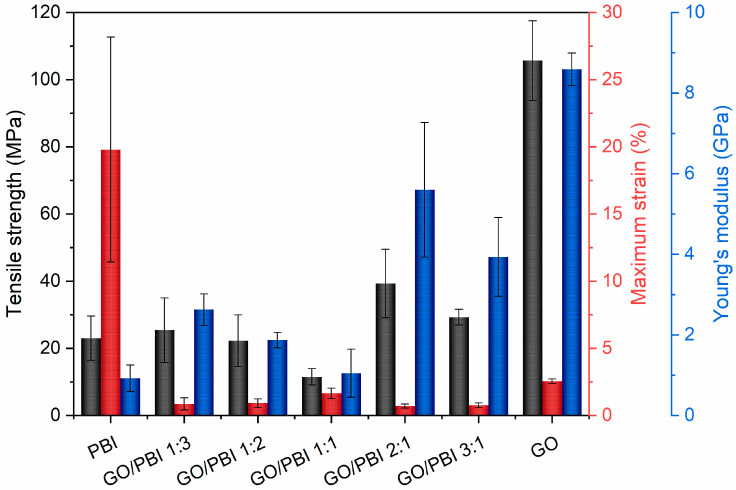
Values of tensile strength (black), maximum strain (red), and Young’s modulus (blue) extrapolated from tensile tests performed on the GO/PBI X:Y composites, pure GO, and pure PBI membranes.

The average tensile strengths (TS), maximum strains (MS), and Young’s moduli (YM) of the investigated GO/PBI X:Y samples, pure GO, and pure PBI are displayed in Figure 6. The aim of the tensile tests was the comprehension of the effects of the different GO-to-PBI mass ratios on the mechanical properties of the composite membranes. Pure PBI and pure GO showed expected values of TS (23.0 ± 6.6 and 105.7 ± 11.9 MPa, respectively) and YM (0.9 ± 0.3 and 8.6 ± 0.4 GPa, respectively) [22,30,32,33,39,50]. The main information that was extracted from the experimental outcomes concerned the enhancement of mechanical resistance and elasticity of the composite samples with respect to PBI, favored by the predominance of GO. The best values were derived for GO/PBI 2:1 (TS of 39.3 ± 10.2 MPa, YM of 5.6 ± 1.7 GPa) and GO/PBI 3:1 (TS of 29.3 ± 2.3 MPa, YM of 3.9 ± 1.0 GPa), due to the positive influence of the GO framework in which the polymer was homogeneously dispersed.

Nevertheless, an overall embrittlement of the final products was observed in parallel to the reinforcement promoted by mutual π-π interactions, as also discussed in other studies regarding PBI-based materials [32,46]. MS values of the composite membranes were up to two orders of magnitude lower than pure PBI (19.8 ± 8.4%), and also lower than pure GO (2.6 ± 0.2%). The complete absence of a plastic region in the extrapolated stress–strain curves (Figure S2) justified the classification of the GO/PBI X:Y composite membranes as rigid materials. Probably, stacked layers of alternated constituents were unable to sustain a prolonged reciprocal sliding, causing the samples to prematurely fracture. However, the presence of possible micro-defects acting as sites of stress concentration, generated during the trimming of strips from the as-prepared membranes, could be presumed as much as one of the factors contributing to the low MS values detected during tensile tests on the GO/PBI X:Y composites.

### 3.4. Ion Exchange Capacity and Proton Conductivity

**Figure 7:**
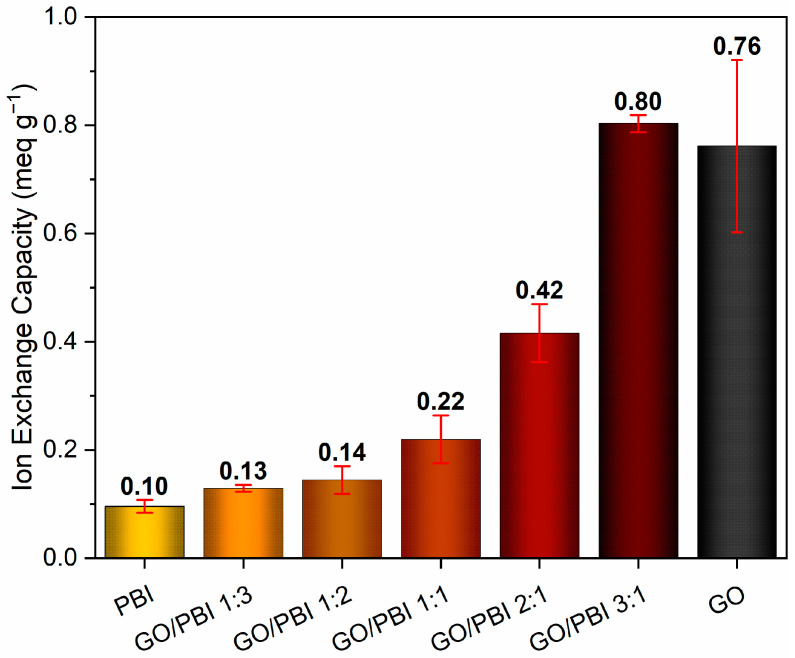
Ion exchange capacity values of the GO/PBI X:Y composites, pure GO, and pure PBI membranes.

Figure 7 shows the average values of ion exchange capacity determined for the GO/PBI X:Y composite membranes together with those of benchmark GO and PBI samples. An evident increasing trend due to the influence of higher GO contents can be appreciated, in agreement with similar results discussed in other works [33,51]. The poor ion exchange tendency (0.10 ± 0.01 meq g^−1^) of the virgin polymeric membrane, equivalent to the outcome reported by Mondal et al. [51], may be imputed to the scarcity of mobile protons within its structure. The combination with higher concentrations of GO was beneficial, due to the introduction of sites able to exchange protons, i.e., carboxyl (–COOH) and hydroxyl (–OH) functionalities, in the structure of the composite membranes. The one characterized by the lowest GO mass content (GO/PBI 1:3) provided an IEC performance of 0.13 ± 0.01 meq g^−1^, a 30% improvement with respect to pure PBI. Then, further improvements were progressively recorded up to GO/PBI 3:1, whose IEC value (0.80 ± 0.02 meq g^−1^) resulted to be the highest among the studied samples, eight times higher than pure PBI, and comparable with that of pure GO (0.76 ± 0.16 meq g^−1^). The discussed IEC trend recommended again an optimal reciprocal dispersion of PBI and GO up to the achievement of the alternated stacked structure proposed in Section 3.2. In this fashion, PBI moieties did not interfere with the availability of proton-exchanging sites of GO, allowing for higher IEC values at higher GO mass contents.

Table 3 reports the proton conductivities of the GO/PBI X:Y composites membranes, of pure GO, and of pure PBI computed after EIS tests. This characterization was specifically performed to achieve the third and last aim of this work, i.e., to verify if the composite materials with innovative GO-to-PBI mass ratios might be employed as proton conductors in electrochemical devices working between 80 and 120 °C.

Proton conductivities in the order of 10^–2^ S cm^−1^ were determined for all the composite membranes, with negligible variability. On the basis of the existing literature about materials presented as potential proton exchange membranes, which provided comparable, if not lower, performances [7,23,34,46,52], these results can be considered as promising, especially in the case of GO/PBI 2:1 and GO/PBI 3:1 (0.0464 S cm^−1^ and 0.0451 S cm^−1^ at 100 °C, respectively). In general, increasing contents of GO in the composite membranes corresponded to higher proton conductivities. This trend was attributed to the larger presence of oxygenated functionalities, able to operate as proton-hopping sites, delivered by larger quantities of GO [34,46,52]. Moreover, the hydrophilic nature of GO could have also conferred a better water retention tendency to the GO/PBI X:Y samples, further promoting the transport of protons between the electrodes due to water clustering. Nevertheless, the different thicknesses of the GO/PBI X:Y composites, related to the different quantities of GO and PBI used to guarantee a specific X:Y mass ratio, could have influenced the extrapolated outcomes by flattening the actual effects of larger GO mass contents on the ability of the samples to transport protons [53]. It is also worth noticing that the improvement of proton conductivity with respect to pristine PBI was possible by only sacrificing the plasticity of the polymer.

Temperature slightly affected the measured proton conductivities as well. Moderately higher values were determined up to 100 °C for the GO/PBI X:Y composites characterized by high GO-to-PBI mass ratios. An influence of this kind was expected, since temperature is renowned for facilitating proton mobility [7,37,39]. On the contrary, a performance worsening occurred at 120 °C. Two correlated phenomena may have accounted for this experimental outcome. The first was the possible partial reduction of the GO sheets within the composite membranes. The second was the minimized availability of water clusters, due to both a facilitated evaporation at the highest examined temperature and to the fewer oxygen-containing moieties around which water can amass. The comparable drop displayed by bare GO was believed to be a confirmation of such issues.

Two further details deserve to be commented on. To begin, the remarkable IEC of GO/PBI 3:1 did not correspond to proton conductivity equivalent to pristine GO. The explanation may be found in the difference existing between the static exchange of ions in the solution during the IEC experiments and the dynamic motion of the protons in the EIS tests. The static exchange of ions was possible due to the carboxyl (–COOH) and hydroxyl (–OH) moieties decorating the GO flakes. Conversely, the alignment of alternated GO and PBI layers in the composite could have prevented the generation of continuous pathways through which proton transport can be exerted. As a last consideration, GO/PBI 1:1 showed the worst proton conductivities at every temperature. This anomaly could be imputed to its GO-to-PBI mass ratio, which implicated both the highest thickness (84 ± 16 µm) due to the employed quantities of GO and PBI (Table 1) and a total absence of disproportion between GO and PBI. As a consequence of this last aspect, uninterrupted transport channels within the membrane, essential for proton conduction, were likely impossible to constitute. Combining this result with the anomalous XRD pattern discussed in Section 3.2 and the negative mechanical properties highlighted in Figure 6, a 1:1 GO-to-PBI mass ratio appeared to be disadvantageous.

A comparison of tensile strength, ion exchange capacity, and proton conductivity values of GO/PBI 2:1, GO/PBI 3:1, and other state-of-the-art materials investigated in previous works is proposed in Table 4. The potentiality of GO/PBI X:Y composites with high GO-to-PBI mass ratios for an application in electrochemical devices is supported by their good IEC and proton conductivity, albeit an improvement in mechanical properties would be beneficial.

## 4. Conclusions

In this work, an easy, safe, and reproducible procedure for the preparation of self-assembling GO/PBI composites, characterized by large relative quantities of both components, was developed. Five different GO/PBI X:Y membranes were fabricated, accordingly, by employing GO-to-PBI mass ratios of 1:3, 1:2, 1:1, 2:1, and 3:1, i.e., with GO mass percentages one order of magnitude higher than the ones reported in the literature for comparable materials. An extensive investigation of the prepared samples was performed by means of scanning electron microscopy (SEM), X-ray diffraction (XRD), thermogravimetric analysis (TGA), and tensile tests. All the GO/PBI X:Y composite membranes demonstrated to be stable and uniform. The cohesion and homogenous reciprocal dispersion among GO layers and PBI strands were suggested by SEM analysis, whereas XRD patterns suggested the constitution of an alternated stacked framework in which the primary role was covered by mutual π-π interactions among the benzimidazole rings of PBI and the aromatic regions of GO. Thermal behavior of GO was enhanced due to the combination with thermally stable PBI chains. The presence of GO flakes helped to improve the tensile strength and Young’s modulus of the composites with respect to pure PBI, at the cost of a sensible loss in the corresponding maximum strains.

Moreover, ion exchange capacity (IEC) evaluation and electrochemical impedance spectroscopy (EIS) tests were executed to evaluate, in a preliminary fashion, the suitability of the GO/PBI X:Y composites to work as proton exchange membranes in electrochemical devices. Due to the positive effect of the hydrophilic oxygenated functional groups of GO, the results were overall encouraging. In particular, GO/PBI 2:1 (IEC of 0.42 meq g^−1^, proton conductivity of 0.0464 S cm^−1^ at 100 °C) and GO/PBI 3:1 (IEC of 0.80 meq g^−1^, proton conductivity of 0.0451 S cm^−1^ at 100 °C) provided equivalent or superior performances with respect to similar state-of-the-art materials proposed in the literature.

## Figures and Tables

**Table 1:** Preparation quantities of GO, PBI, and corresponding solutions, X:Y mass ratios, and average thicknesses of the GO/PBI X:Y composite membranes. Data of the benchmark GO and PBI membranes are reported for comparison.

Sample	GO Mass (g)	PBI Mass (g)	X:Y Mass Ratio	Thickness (µm)
GO/PBI 1:3	0.05	0.15	1:3	60 ± 7
GO/PBI 1:2	0.075	0.15	1:2	63 ± 13
GO/PBI 1:1	0.15	0.15	1:1	84 ± 16
GO/PBI 2:1	0.15	0.075	2:1	45 ± 7
GO/PBI 3:1	0.15	0.05	3:1	51 ± 7
GO	0.15	–	–	4 ± 0.2
PBI	–	0.15	–	58 ± 7

**Table 2:** Interplanar distances of the GO/PBI X:Y composite, pure GO, and pure PBI membranes.

Sample	Reflection Position (°)	Interplanar Distance (nm)
GO/PBI 1:3	8.54	1.03
GO/PBI 1:2	8.52	1.04
GO/PBI 1:1	7.88	1.12
GO/PBI 2:1	8.48	1.04
GO/PBI 3:1	8.88	0.99
GO	10.78	0.82
PBI	24.28	0.37

**Table 3:** Proton conductivity values of GO/PBI X:Y composites, pure GO, and pure PBI from EIS tests executed at 60, 80, 100, and 120 °C.

Sample	Temperature (°C)			
	60	80	100	120
	Proton Conductivity (S cm^−1^)			
GO/PBI 1:3	0.0337 (±5.5 × 10^–5^)	0.0338 (±5.7 × 10^–5^)	0.0337 (±1.5 × 10^–4^)	0.0337 (±8.1 × 10^–5^)
GO/PBI 1:2	0.0343 (±4.2 × 10^–5^)	0.0344 (±4.8 × 10^–5^)	0.0344 (±3.2 × 10^–6^)	0.0344 (±1.9 × 10^–5^)
GO/PBI 1:1	0.0263 (±2.6 × 10^–5^)	0.0267 (±1.2 × 10^–5^)	0.0266 (±1.4 × 10^–5^)	0.0263 (±5.3 × 10^–5^)
GO/PBI 2:1	0.0457 (±7.5 × 10^–5^)	0.0463 (±6.6 × 10^–5^)	0.0464 (±4.2 × 10^–5^)	0.0456 (±7.3 × 10^–5^)
GO/PBI 3:1	0.0442 (±4.7 × 10^–5^)	0.0451 (±6.1 × 10^–5^)	0.0451 (±1.0 × 10^–4^)	0.0438 (±5.4 × 10^–5^)
GO	0.1201 (±1.5 × 10^–3^)	0.1966 (±3.6 × 10^–3^)	0.2100 (±1.3 × 10^–4^)	0.0990 (±2.5 × 10^–4^)
PBI	0.0321 (±1.9 × 10^–6^)	0.0321 (±5.0 × 10^–5^)	0.0322 (±3.2 × 10^–5^)	0.0322 (±1.3 × 10^–4^)

**Table 4:** Comparison of the properties of GO/PBI 2:1 and GO/PBI 3:1 with other state-of-the-art GO- and PBI-related materials reported in the literature.

Material	Tensile Strength (MPa)	IEC (meq g^−1^)	Proton Conductivity (S cm^−1^)	Reference
C5F6-PBI	107	–	≈0.03 @ 120 °C	[7]
CBOPBI@MOF40%	50.5	–	≈0.09 @ 120 °C	[8]
PBI-ZIF8 (10.0 wt%)	4.6	–	≈0.025 @ 140 °C	[31]
PBI-SGO 6 wt%	28.2	2.44	≈0.02 @ 120 °C	[33]
PBI/sGO-2	11	–	0.099 @ 140 °C	[34]
Py-PBI/1.5% PGO	4.65	–	0.0764 @ 140 °C	[36]
PBI/PGO-1.5%	167.8	–	≈0.007 @ 100 °C	[37]
PBI/ImGO_0.5	219.2	–	0.0341 @ 100 °C	[46]
PBI/30%-SNW-1	4	–	≈0.1 @ 120 °C	[49]
5% SGO/SPBI	28.6	1.00	0.018 @ 25 °C	[51]
GO/PBI 2:1	39.28	0.42	0.0464 @ 100 °C	This work
GO/PBI 3:1	29.28	0.80	0.0451 @ 100 °C	This work

## Data Availability

Data are contained within the article. All data are available upon reasonable request from the corresponding author.

## Supplementary Materials

The following supporting information can be downloaded at: https://www.mdpi.com/article/10.3390/membranes13040428/s1, Figure S1: Sketch of the alternated stacked framework proposed to describe the structure of the GO/PBI X:Y composite membranes; Figure S2: Stress–strain curves of (a) GO/PBI 1:3; (b) GO/PBI 1:2; (c) GO/PBI 1:1; (d) GO/PBI 2:1; (e) GO/PBI 3:1. For comparison purposes, the mechanical behaviors of (f) pure GO and (g) pure PBI are reported as well.

## References

[1] Escorihuela J., Olvera-Mancilla J., Alexandrova L., del Castillo L.F., Compañ V. (2020). Recent Progress in the Development of Composite Membranes Based on Polybenzimidazole for High Temperature Proton Exchange Membrane (PEM) Fuel Cell Applications. Polymers.

[2] Quartarone E., Angioni S., Mustarelli P. (2017). Polymer and Composite Membranes for Proton-Conducting, High-Temperature Fuel Cells: A Critical Review. Materials.

[3] Kamaroddin M.F.A., Sabli N., Tuan Abdullah T.A., Siajam S.I., Abdullah L.C., Abdul Jalil A., Ahmad A. (2021). Membrane-Based Electrolysis for Hydrogen Production: A Review. Membranes.

[4] Sun X., Simonsen S., Norby T., Chatzitakis A. (2019). Composite Membranes for High Temperature PEM Fuel Cells and Electrolysers: A Critical Review. Membranes.

[5] Wang Y., Shi Z., Fang J., Xu H., Yin J. (2011). Graphene oxide/polybenzimidazole composites fabricated by a solvent-exchange method. Carbon N. Y..

[6] Sulaiman R.R.R., Walvekar R., Khalid M., Wong W.Y., Jagadish P. (2020). Recent Progress in the Development of Aromatic Polymer-Based Proton Exchange Membranes for Fuel Cell Applications. Polymers.

[7] Wang L., Liu Z., Liu Y., Wang L. (2019). Crosslinked polybenzimidazole containing branching structure with no sacrifice of effective N-H sites: Towards high-performance high-temperature proton exchange membranes for fuel cells. J. Memb. Sci..

[8] Wu Y., Liu X., Yang F., Lee Zhou L., Yin B., Wang P., Wang L. (2021). Achieving high power density and excellent durability for high temperature proton exchange membrane fuel cells based on crosslinked branched polybenzimidazole and metal-organic frameworks. J. Memb. Sci..

[9] Melchior J.-P., Majer G., Kreuer K.-D. (2017). Why do proton conducting polybenzimidazole phosphoric acid membranes perform well in high-temperature PEM fuel cells?. Phys. Chem. Chem. Phys..

[10] Jeong Y.H., Oh K., Ahn S., Kim N.Y., Byeon A., Park H.-Y., Lee S.Y., Park H.S., Yoo S.J., Jang J.H. (2017). Investigation of electrolyte leaching in the performance degradation of phosphoric acid-doped polybenzimidazole membrane-based high temperature fuel cells. J. Power Sources.

[11] Chen Y., Azizi K., Zhang W., Aili D., Primdahl S., Cleemann L.N., Hjuler H.A., Li Q. (2022). Feasibility of using thin polybenzimidazole electrolytes in high-temperature proton exchange membrane fuel cells. Int. J. Hydrog. Energy.

[12] Wang X., Jayaweera P., Alrasheed R., Aljlil S., Alyousef Y., Alsubaei M., AlRomaih H., Jayaweera I. (2018). Preparation of Polybenzimidazole Hollow-Fiber Membranes for Reverse Osmosis and Nanofiltration by Changing the Spinning Air Gap. Membranes.

[13] Oxley A., Livingston A.G. (2022). Anti-fouling membranes for organic solvent nanofiltration (OSN) and organic solvent ultrafiltration (OSU): Graft modified polybenzimidazole (PBI). J. Memb. Sci..

[14] Kallem P., Charmette C., Drobek M., Julbe A., Mallada R., Pina M. (2018). Exploring the Gas-Permeation Properties of Proton-Conducting Membranes Based on Protic Imidazolium Ionic Liquids: Application in Natural Gas Processing. Membranes.

[15] Singh R.P., Dahe G.J., Dudeck K.W., Berchtold K.A. (2020). Macrovoid-free high performance polybenzimidazole hollow fiber membranes for elevated temperature H_2_/CO_2_ separations. Int. J. Hydrog. Energy.

[16] Lee Y., Kim S., Maljusch A., Conradi O., Kim H.-J., Jang J.H., Han J., Kim J., Henkensmeier D. (2019). Polybenzimidazole membranes functionalised with 1-methyl-2-mesitylbenzimidazolium ions via a hexyl linker for use in vanadium flow batteries. Polymer.

[17] Dai Q., Xing F., Liu X., Shi D., Deng C., Zhao Z., Li X. (2022). High-performance PBI membranes for flow batteries: From the transport mechanism to the pilot plant. Energy Environ. Sci..

[18] Üregen N., Pehlivanoğlu K., Özdemir Y., Devrim Y. (2017). Development of polybenzimidazole/graphene oxide composite membranes for high temperature PEM fuel cells. Int. J. Hydrog. Energy.

[19] Hooshyari K., Rezania H., Vatanpour V., Salarizadeh P., Askari M.B., Beydaghi H., Enhessari M. (2020). High temperature membranes based on PBI/sulfonated polyimide and doped-perovskite nanoparticles for PEM fuel cells. J. Memb. Sci..

[20] Sean N.A., Leaw W.L., Abouzari-Lotf E., Nur H. (2021). Magnetic field-induced alignment of polybenzimidazole microstructures to enhance proton conduction. J. Chin. Chem. Soc..

[21] Perry A.K., More L.K., Andrew Payzant E., Meisner R.A., Sumpter B.G., Benicewicz B.C. (2014). A comparative study of phosphoric acid-doped m -PBI membranes. J. Polym. Sci. Part B Polym. Phys..

[22] Kamaroddin M.F.A., Sabli N., Nia P.M., Abdullah T.A.T., Abdullah L.C., Izhar S., Ripin A., Ahmad A. (2020). Phosphoric acid doped composite proton exchange membrane for hydrogen production in medium-temperature copper chloride electrolysis. Int. J. Hydrog. Energy.

[23] Diaz-Abad S., Fernández-Mancebo S., Rodrigo M.A., Lobato J. (2022). Characterization of PBI/Graphene Oxide Composite Membranes for the SO2 Depolarized Electrolysis at High Temperature. Membranes.

[24] Sun C.-Y., Negro E., Nale A., Pagot G., Vezzù K., Zawodzinski T.A., Meda L., Gambaro C., Di Noto V. (2021). An efficient barrier toward vanadium crossover in redox flow batteries: The bilayer [Nafion/(WO_3_)x] hybrid inorganic-organic membrane. Electrochim. Acta.

[25] Okonkwo P.C., Ben Belgacem I., Emori W., Uzoma P.C. (2021). Nafion degradation mechanisms in proton exchange membrane fuel cell (PEMFC) system: A review. Int. J. Hydrog. Energy.

[26] Shiva Kumar S., Himabindu V. (2019). Hydrogen production by PEM water electrolysis—A review. Mater. Sci. Energy Technol..

[27] Maity S., Singha S., Jana T. (2015). Low acid leaching PEM for fuel cell based on polybenzimidazole nanocomposites with protic ionic liquid modified silica. Polymer.

[28] Heinzl C., Ossiander T., Gleich S., Scheu C. (2015). Transmission electron microscopy study of silica reinforced polybenzimidazole membranes. J. Memb. Sci..

[29] Devrim Y., Devrim H., Eroglu I. (2016). Polybenzimidazole/SiO2 hybrid membranes for high temperature proton exchange membrane fuel cells. Int. J. Hydrog. Energy.

[30] Kuo Y.-J., Lin H.-L. (2018). Effects of mesoporous fillers on properties of polybenzimidazole composite membranes for high-temperature polymer fuel cells. Int. J. Hydrog. Energy.

[31] Eren E.O., Özkan N., Devrim Y. (2022). Preparation of polybenzimidazole/ZIF-8 and polybenzimidazole/UiO-66 composite membranes with enhanced proton conductivity. Int. J. Hydrog. Energy.

[32] Zhao B., Cheng L., Bei Y., Wang S., Cui J., Zhu H., Li X., Zhu Q. (2017). Grafted polybenzimidazole copolymers bearing polyhedral oligosilsesquioxane pendant moieties. Eur. Polym. J..

[33] Yusoff Y.N., Loh K.S., Wong W.Y., Daud W.R.W., Lee T.K. (2020). Sulfonated graphene oxide as an inorganic filler in promoting the properties of a polybenzimidazole membrane as a high temperature proton exchange membrane. Int. J. Hydrog. Energy.

[34] Devrim Y., Bulanık Durmuş G.N. (2022). Composite membrane by incorporating sulfonated graphene oxide in polybenzimidazole for high temperature proton exchange membrane fuel cells. Int. J. Hydrog. Energy.

[35] Sulaiman R.R.R., Walvekar R., Wong W.Y., Khalid M., Pang M.M. (2022). Proton Conductivity Enhancement at High Temperature on Polybenzimidazole Membrane Electrolyte with Acid-Functionalized Graphene Oxide Fillers. Membranes.

[36] Abouzari-Lotf E., Zakeri M., Nasef M.M., Miyake M., Mozarmnia P., Bazilah N.A., Emelin N.F., Ahmad A. (2019). Highly durable polybenzimidazole composite membranes with phosphonated graphene oxide for high temperature polymer electrolyte membrane fuel cells. J. Power Sources.

[37] Zhao X., Nan B., Lu Y., Zhao C., Xu S. (2021). Phosphorylated graphene oxide-reinforced polybenzimidazole composite membrane for high-temperature proton exchange membrane fuel cell. J. Polym. Res..

[38] Basso Peressut A., Di Virgilio M., Bombino A., Latorrata S., Muurinen E., Keiski R.L., Dotelli G. (2022). Investigation of Sulfonated Graphene Oxide as the Base Material for Novel Proton Exchange Membranes. Molecules.

[39] Di Virgilio M., Basso Peressut A., Latorrata S., Mariani M., Dotelli G. (2022). Graphene oxide-naphthalene sulfonate blends as possible proton exchange membranes. Solid State Ion..

[40] Yuan X.-Z., Song C., Wang H., Zhang J. (2010). EIS Equivalent Circuits. Electrochemical Impedance Spectroscopy in PEM Fuel Cells.

[41] Cai Y., Yue Z., Teng X., Xu S. (2018). Phosphoric Acid Doped Crosslinked Polybenzimidazole/Modified Graphene Oxide Composite Membranes for High Temperature Proton Exchange Membrane Applications. J. Electrochem. Soc..

[42] Wang Y., Feng K., Ding L., Wang L., Han X. (2020). Influence of solvent on ion conductivity of polybenzimidazole proton exchange membranes for vanadium redox flow batteries. Chinese J. Chem. Eng..

[43] Figoli A., Marino T., Simone S., Di Nicolò E., Li X.-M., He T., Tornaghi S., Drioli E. (2014). Towards non-toxic solvents for membrane preparation: A review. Green Chem..

[44] Latorrata S., Cristiani C., Basso Peressut A., Brambilla L., Bellotto M., Dotelli G., Finocchio E., Gallo Stampino P., Ramis G. (2020). Reduced Graphene Oxide Membranes as Potential Self-Assembling Filter for Wastewater Treatment. Minerals.

[45] Haynes W.M. (2014). Section 3—Physical Constants of Organic Compounds. CRC Handbook of Chemistry and Physics.

[46] Kim J., Kim K., Ko T., Han J., Lee J.-C. (2021). Polybenzimidazole composite membranes containing imidazole functionalized graphene oxide showing high proton conductivity and improved physicochemical properties. Int. J. Hydrog. Energy.

[47] Ahn M., Liu R., Lee C., Lee W. (2019). Designing Carbon/Oxygen Ratios of Graphene Oxide Membranes for Proton Exchange Membrane Fuel Cells. J. Nanomater..

[48] Imran M.A., Li T., Wu X., Yan X., Khan A.-S., He G. (2020). Sulfonated polybenzimidazole/amine functionalized titanium dioxide (sPBI/AFT) composite electrolyte membranes for high temperature proton exchange membrane fuel cells usage. Chin. J. Chem. Eng..

[49] Cheng G., Li Z., Qu E., Ren S., Han D., Xiao M., Wang S., Meng Y. (2022). N-H group-rich dendrimer doped polybenzimidazole composite membrane with consecutive proton transportation channels for HT-PEMFCs. Electrochim. Acta.

[50] Gong T., Van Lam D., Liu R., Won S., Hwangbo Y., Kwon S., Kim J., Sun K., Kim J.-H., Lee S.-M. (2015). Thickness Dependence of the Mechanical Properties of Free-Standing Graphene Oxide Papers. Adv. Funct. Mater..

[51] Mondal S., Papiya F., Ash S.N., Kundu P.P. (2021). Composite membrane of sulfonated polybenzimidazole and sulfonated graphene oxide for potential application in microbial fuel cell. J. Environ. Chem. Eng..

[52] Xu C., Cao Y., Kumar R., Wu X., Wang X., Scott K. (2011). A polybenzimidazole/sulfonated graphite oxide composite membrane for high temperature polymer electrolyte membrane fuel cells. J. Mater. Chem..

[53] Sun C., Zhang H. (2019). Investigation of Nafion series membranes on the performance of iron-chromium redox flow battery. Int. J. Energy Res..

