# Supplementary material for: Study of Innovative GO/PBI Composites as Possible Proton Conducting Membranes for Electrochemical Devices
Source: Membranes (Basel). 2023 Apr 13;13(4):428. doi: 10.3390/membranes13040428 (PMC10143660; doi:10.3390/membranes13040428)
Supplement: Supplementary file 1 [file membranes-13-00428-s001.zip › membranes-2219409-supplementary.pdf]

Study of innovative GO/PBI composites as possible proton conducting membranes for electrochemical devices

Matteo Di Virgilio, Andrea Basso Peressut *, Angelo Pontoglio, Saverio Latorrata * and Giovanni Dotelli

Department of Chemistry, Materials and Chemical Engineering "Giulio Natta", Politecnico di Milano, Piazza Leonardo da Vinci 32, 20133 Milano, Italy; matteo.divirgilio@polimi.it (M.D.V.); angelo.pontoglio@mail.polimi.it (A.P.); giovanni.dotelli@polimi.it (G.D.)
* Correspondence: andreastefano.basso@polimi.it (A.B.P), saverio.latorrata@polimi.it (S.L); Tel.: +39-02-2399-3190 (A.B.P. & S.L.)

Supplementary materials

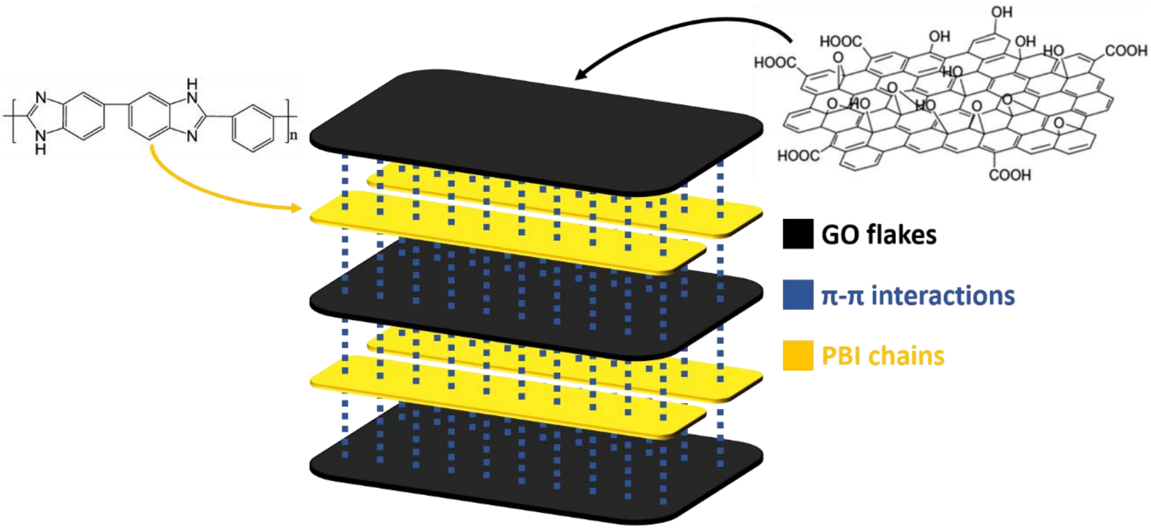

Figure S1. Sketch of the alternated stacked framework proposed to describe the structure of the GO/PBI X:Y composite membranes.

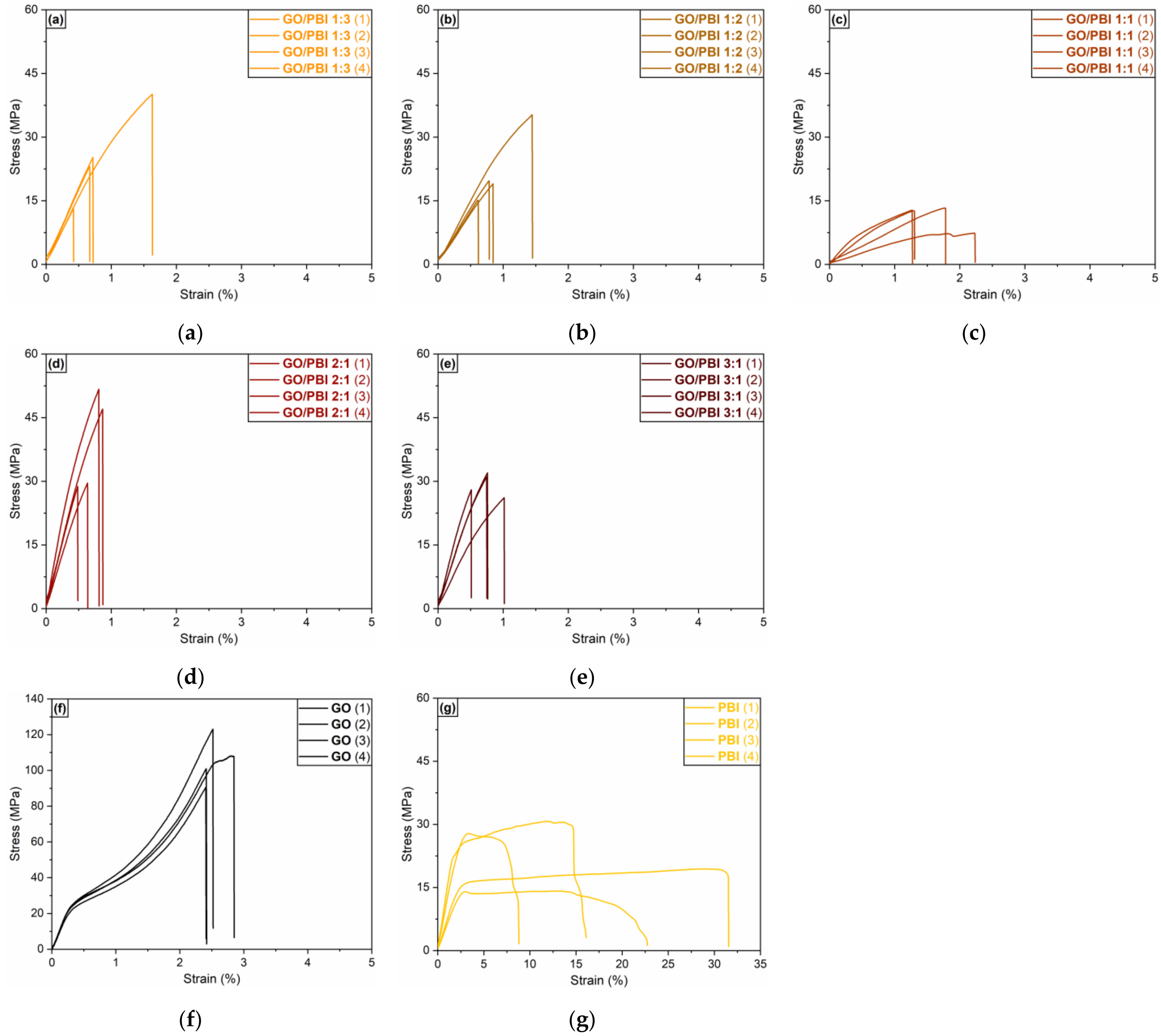

Figure S2. Stress-strain curves of (**a**) GO/PBI 1:3; (**b**) GO/PBI 1:2; (**c**) GO/PBI 1:1; (**d**) GO/PBI 2:1; (**e**) GO/PBI 3:1. For comparison purposes, the mechanical behaviors of (**f**) pure GO and (**g**) pure PBI are reported as well.
